# W-transform for exponential stability of second order delay differential equations without damping terms

**DOI:** 10.1186/s13660-017-1296-0

**Published:** 2017-01-17

**Authors:** Alexander Domoshnitsky, Abraham Maghakyan, Leonid Berezansky

**Affiliations:** 1 Department of Mathematics, Ariel University, Ariel, Israel; 2 Department of Mathematics, Ben-Gurion University of the Negev, Beer-Sheva, 84105 Israel

**Keywords:** 34K20, 34K06, 34K25, second order delay differential equations, W-method, exponential stability

## Abstract

In this paper a method for studying stability of the equation $x^{\prime \prime }(t)+\sum_{i=1}^{m}a_{i}(t)x(t- \tau_{i}(t))=0$ not including explicitly the first derivative is proposed. We demonstrate that although the corresponding ordinary differential equation $x^{\prime \prime}(t)+\sum_{i=1}^{m}a_{i}(t)x(t)=0$ is not exponentially stable, the delay equation can be exponentially stable.

## Introduction

In this paper we apply the W-method to obtain explicit conditions for exponential stability of linear second order delay differential equations. The method consists in a transformation of a given differential equation to an operator equation by the substitution

$$x(t)= \int_{0}^{t} W(t,s) z(s)\,ds, $$

where $W(t,s)$ is the Cauchy function for some known exponentially stable equation.

The main object of this paper is the second order delay differential equation

1.1 $$ x^{\prime \prime }(t)+\sum_{i=1}^{m}a_{i}(t)x \bigl(t-\tau_{i}(t) \bigr)- \sum_{i=1}^{m}b_{i}(t)x \bigl(t-\theta_{i}(t) \bigr)=f(t),\quad t\in [ 0,+\infty), $$

with a corresponding initial function defining what should be put in the equation instead of $x(t-\tau_{i}(t))$ when $t-\tau_{i}(t)<0$ or $x(t-\theta_{i}(t))$ when $t-\theta_{i}(t)<0$. For simplicity and without loss of generality we can consider the zero initial function

1.2 $$ x(\xi)=0, \quad \text{for } \xi < 0. $$

Concerning the coefficients, delays, and function *f* we assume that *f*, $a_{i}$, $b_{i}$, *φ*, $\tau_{i}$, $\theta_{i}$ ($i=1,\ldots,m$) are measurable essentially bounded functions $[0,+\infty)\rightarrow (-\infty,+ \infty)$, and $\tau_{i}(t)\geq 0$, $\theta_{i}(t)\geq 0$ for $t\geq 0$. We understand by a solution of equation (1.1), (1.2) a function $x:[0,+\infty)\rightarrow (-\infty,+\infty)$ with absolutely continuous on every finite interval derivative $x^{\prime }$ and essentially bounded second derivative $x^{\prime \prime }$ which satisfies this equation almost everywhere.

Various applications of equation (1.1) and its generalizations can be found, for example, in the theory of self-excited oscillations, in oscillation processes in a vacuum tube, in dynamics of an auto-generator, in description of processes of in-feed grinding and cutting (see [1]); on position control in mechanical engineering, on electromechanical systems, and on combustion engines [2]. The problem of stabilizing the rolling of a ship by the activated tanks method in which ballast water is pumped from one position to another was reduced in [3] to analysis of stability of the second order delay equation.

Asymptotic properties of second order equations without damping term were studied in ([4], Chapter III, Section 16, pp.105-106), where instability of the equation

$$x^{\prime \prime }(t)+bx(t-\tau)=0, $$

for every pair of positive constants *b* and *τ* was obtained. Conditions of unboundedness of solutions to the equation

$$x^{\prime \prime }(t)+\sum_{i=1}^{m}b_{i}(t)x \bigl(t-\tau_{i}(t) \bigr)=0, $$

with variable coefficients and delays were obtained in [5]. The condition $\int _{0}^{\infty }\tau (t)\,dt< \infty $ is necessary and sufficient for the boundedness of all solutions to the equation

$$x^{\prime \prime }(t)+bx \bigl(t-\tau (t) \bigr)=0 $$

(see [5]). Results as regards the boundedness of solutions for vanishing delays ($\tau_{i}(t)\rightarrow 0$ for $t\rightarrow \infty$) and as regards asymptotic representations of solutions were obtained in [6, 7], see also [4], Chapter III, Section 16. Boundedness of solutions for equations with advanced arguments ($\tau_{i}(t)\leq 0$) was studied in [8]. First results on the exponential stability for the equation $x^{\prime \prime }(t)+ax(t)-bx(t-\tau)=0$ with constant coefficients and delay were obtained in [9–11]. First results on the exponential stability of the second order equation (1.1) without damping term and with variable coefficients and delays were obtained recently in [12]. All results of [12] concern only the case of a non-oscillation equation,

1.3 $$\begin{aligned}& \begin{aligned} &x^{\prime \prime }(t)+\sum _{i=1}^{m}a_{i}(t)x \bigl(t- \tau_{i}(t) \bigr)- \sum_{i=1}^{m}b_{i}(t)x \bigl(t-\theta_{i}(t) \bigr)=0,\quad t\in [ 0,+\infty), \\ &x(\xi)=0,\quad \text{for } \xi < 0 \end{aligned} \end{aligned}$$

and cannot be efficiently applied to the oscillation case. Our present paper fills this gap.

Our method reduces the study of stability of equations without damping term to the study of stability of corresponding equations with damping term. The stability of autonomous delay differential equations of the second order with damping terms was studied in [1, 9, 10], which in the case of delay differential equations apply quasi-polynomials and not polynomials as in the case of ordinary differential equations. That is why a special approach for the analysis of the characteristic equations is used [13]. The technique of the Lyapunov functions was used in the works [14–16] and the technique of the fixed point theorems in [17]. The technique of non-oscillation and positivity of the Cauchy functions for studying stability of delay equations was proposed [18] and then developed in [19].

The general solution of equation (1.1), (1.2) can be represented in the form [20]

1.4 $$ x(t)= \int _{0}^{t}C(t,s)f(s)\,ds+x_{1}(t)x(0)+x_{2}(t)x^{\prime }(0), $$

where $x_{1}(t)$, $x_{2}(t)$ are two solutions of homogeneous equation (1.3), (1.2) satisfying the conditions

1.5 $$ x_{1}(0)=1,\qquad x_{1}^{\prime }(0)=0, \qquad x_{2}(0)=0,\qquad x_{2}^{\prime }(0)=1, $$

the kernel $C(t,s)$ in this representation is called the Cauchy function (fundamental function in other terminology) of equation (1.1).

Consider equation (1.3) with the following initial conditions:

1.6 $$ x(\xi)=\varphi (\xi),\quad \xi < t_{0},\qquad x(t_{0})=x_{0}, \qquad x^{\prime }(t _{0})=x_{0}^{\prime }. $$

Let us formulate several definitions concerning stability.

### Definition 1

Equation (1.3) is uniformly exponentially stable if there exist $N>0$ and $\alpha >0$, such that the solution of (1.3), (1.6) satisfies the estimates

1.7 $$ \bigl\vert x(t) \bigr\vert \leq Ne^{-\alpha (t-t_{0})}\sup _{t< t_{0}} \bigl\vert \varphi(\xi) \bigr\vert ,\qquad \bigl\vert x^{\prime }(t) \bigr\vert \leq Ne^{- \alpha (t-t_{0})} \sup_{t< t_{0}} \bigl\vert \varphi (\xi) \bigr\vert ,\quad t_{0}\leq t< +\infty, $$

where *N* and *α* do not depend on $t_{0}$ and initial function *φ*.

### Definition 2

We say that the Cauchy function $C(t,s)$ of equation (1.1) satisfies the exponential estimate if there exist positive $N_{1}$ and $\alpha_{1} $ such that

1.8 $$ \bigl\vert C(t,s) \bigr\vert \leq N_{1}e^{-\alpha_{1} (t-s)}, \qquad \bigl\vert C _{t}^{\prime }(t,s) \bigr\vert \leq N_{1}e^{-\alpha_{1} (t-s)}, \quad 0\leq s\leq t< +\infty. $$

It is well known that for equation (1.1) with bounded delays these two definitions are equivalent [20].

The paper is built as follows. In the first section we describe known results on asymptotic properties of second order delay equations. In Section 2, we formulate the main results of the paper and compare them with known results. Auxiliary assertions can be found in Section 3. The proofs of the assertions, formulated in Section 2, can be found in Section 4.

## Formulation of main results

Let us consider the following ordinary differential equation:

2.1 $$ x^{\prime \prime }(t)+Ax^{\prime }(t)+Bx(t)=0,\quad t\in [ 0,+ \infty), $$

with constant positive coefficients *A* and *B*. We demonstrate that the exponential stability of this equation, under corresponding conditions on coefficients and delays, implies the exponential stability of delay differential equation (1.3). If *x* is a solution of equation (1.1) satisfying (1.2), we can write the equality

2.2 $$\begin{aligned}& \begin{aligned} &x^{\prime \prime }(t)+\sum _{i=1}^{m}A_{i}(t)x^{\prime } \bigl(t- \eta_{i}(t) \bigr)+\sum_{i=1}^{m}B_{i}(t)x \bigl(t-\tau_{i}(t) \bigr)=f(t), \\ &x(\xi)=x^{\prime }(\xi)=0, \quad \xi < 0, \end{aligned} \end{aligned}$$

where $\eta_{i}$ are corresponding measurable functions satisfying inequalities $\theta_{i}(t)\geq \eta_{i}(t)\geq \tau_{i}(t)$,

2.3 $$ A_{i}(t)=b_{i}(t) \bigl( \theta_{i}(t)-\tau_{i}(t) \bigr),\qquad B_{i}(t)=a_{i}(t)-b_{i}(t). $$

Let us denote by

$$A_{i}^{0}=\limsup_{t\rightarrow \infty }\frac{1}{t} \int_{0}^{t} A_{i}(s)\,ds,\qquad B_{i}^{0} =\limsup_{t\rightarrow \infty }\frac{1}{t} \int_{0}^{t} B _{i}(s)\,ds $$

the average values of $A_{i}(t)$ and $B_{i}(t)$, respectively,

2.4 $$\begin{aligned}& \Delta A_{i}(t)=A_{i}^{0}-A_{i}(t), \qquad \Delta B_{i}(t)=B_{i}^{0}-B_{i}(t), \\& \theta_{i}^{\ast }=\mathop{\operatorname{esssup}}_{t\geq 0} \theta_{i}(t),\qquad \theta^{\ast }= \max_{i=1,\ldots,m} \theta_{i}^{\ast }, \\& \tau_{i}^{\ast }=\mathop{\operatorname{esssup}}_{t\geq 0} \tau_{i}(t),\qquad \tau^{\ast }=\max_{i=1,\ldots,m} \tau_{i}^{\ast }. \end{aligned}$$

To connect (2.1) and (2.2) we suppose that the constants *A* and *B* are such that

2.5 $$ A=\sum_{i=1}^{m}A_{i}^{0}, \qquad B=\sum_{i=1}^{m}B_{i}^{0}. $$

### Theorem 1

*Let* $A>0$, $B>0$, $A^{2}>4B$, $\tau_{i}(t)\leq \theta_{i}(t)$ *for* $i=1,\ldots,m$, *and the following inequality be fulfilled*:

2.6 $$\begin{aligned}& \sum_{i=1}^{m} \bigl\vert A_{i}^{0} \bigr\vert \theta_{i}^{ \ast } \biggl\{ \frac{2}{A+\sqrt{A^{2}-4B}} \biggl\{ \frac{A-\sqrt{A ^{2}-4B}}{A+\sqrt{A^{2}-4B}} \biggr\} ^{\frac{A-\sqrt{A^{2}-4B}}{\sqrt{A ^{2}-4B}}}+1 \biggr\} \\& \quad {}+\sum_{i=1}^{m} \bigl\vert \Delta A_{i}(t) \bigr\vert \frac{4}{A+\sqrt{A ^{2}-4B}} \biggl\{ \frac{A-\sqrt{A^{2}-4B}}{A+\sqrt{A^{2}-4B}} \biggr\} ^{\frac{A-\sqrt{A^{2}-4B}}{2\sqrt{A^{2}-4B}}} \\& \quad {}+\sum_{i=1}^{m} \bigl\vert B_{i}^{0} \bigr\vert \tau_{i}^{ \ast } \frac{4}{A+\sqrt{A^{2}-4B}} \biggl\{ \frac{A-\sqrt{A^{2}-4B}}{A+\sqrt{A ^{2}-4B}} \biggr\} ^{\frac{A-\sqrt{A^{2}-4B}}{2\sqrt{A^{2}-4B}}} \\& \quad {}+\sum_{i=1}^{m} \bigl\vert \Delta B_{i}(t) \bigr\vert \frac{1}{B}< 1, \end{aligned}$$

*then the Cauchy function* $C(t,s)$ *of equation* (1.1) *and the fundamental system* $x_{1}(t)$, $x_{2}(t)$ *of equation* (1.3), (1.2) *satisfy an exponential estimate*.

### Theorem 2

*Let* $A>0$, $B>0$, $A^{2}=4B$, $\tau_{i}(t)\leq \theta_{i}(t)$ *for* $i=1,\ldots,m$, *and the following inequality be fulfilled*:

2.7 $$\begin{aligned}& \sum_{i=1}^{m} \bigl\vert A_{i}^{0} \bigr\vert \theta_{i}^{ \ast } \biggl\{ 2+\frac{A}{4}- \biggl( 1-\frac{A}{4} \biggr) \frac{1}{e ^{2}} \biggr\} +\sum_{i=1}^{m} \bigl\vert \Delta A_{i}(t) \bigr\vert \frac{4}{Ae} \\& \quad {} +\sum_{i=1}^{m} \bigl\vert B_{i}^{0} \bigr\vert \tau_{i}^{ \ast } \frac{4}{Ae}+\sum_{i=1}^{m} \bigl\vert \Delta B_{i}(t) \bigr\vert \frac{1}{B}< 1, \end{aligned}$$

*then the Cauchy function* $C(t,s)$ *of equation* (1.1) *and the fundamental system* $x_{1}(t)$, $x_{2}(t)$ *of equation* (1.3), (1.2) *satisfy an exponential estimate*.

### Theorem 3

*Let* $0< A^{2}<4B$, $\tau_{i}(t)\leq \theta_{i}(t)$ *for* $i=1,\ldots,m$, *and the following inequality be fulfilled*:

2.8 $$\begin{aligned}& \sum_{i=1}^{m} \bigl\vert A_{i}^{0} \bigr\vert \theta_{i}^{ \ast } \biggl\{ \frac{2\exp [ -\frac{A}{\sqrt{4B-A^{2}}} ( \pi +2\arctan\frac{\sqrt{4B-A^{2}}}{A} ) ] }{1-\exp [ -\frac{A}{\sqrt{4B-A^{2}}}\pi ] }+1 \biggr\} \\& \quad {}+\sum_{i=1}^{m} \bigl\vert \Delta A_{i}(t) \bigr\vert \frac{2}{ \sqrt{B}}\frac{\exp [ -\frac{A}{\sqrt{4B-A^{2}}} ( \pi + \arctan \frac{\sqrt{4B-A^{2}}}{A} ) ] }{1-\exp [ -\frac{A}{\sqrt{4B-A ^{2}}}\pi ] } \\& \quad {}+\sum_{i=1}^{m} \bigl\vert B_{i}^{0} \bigr\vert \tau_{i}^{ \ast } \frac{2}{\sqrt{B}}\frac{\exp [ -\frac{A}{\sqrt{4B-A ^{2}}} ( \pi +\arctan\frac{\sqrt{4B-A^{2}}}{A} ) ] }{1- \exp [ -\frac{A}{\sqrt{4B-A^{2}}}\pi ] } \\& \quad {} +\sum_{i=1}^{m} \bigl\vert \Delta B_{i}(t) \bigr\vert \frac{1}{B}\frac{1+\exp ( -\frac{\pi A}{\sqrt{4B-A^{2}}} ) }{1-\exp ( -\frac{\pi A}{\sqrt{4B-A^{2}}} ) }< 1, \end{aligned}$$

*then the Cauchy function* $C(t,s)$ *of equation* (1.1) *and the fundamental system* $x_{1}(t)$, $x_{2}(t)$ *of equation* (1.3), (1.2) *satisfy an exponential estimate*.

Let us formulate corollaries for the equation

2.9 $$ x^{\prime \prime }(t)+ax(t-\tau)-bx(t-\theta)=f(t),\quad t\in [ 0,+ \infty), $$

where

2.10 $$ x(\xi)=0, \quad \xi < 0, $$

*f* is a measurable essentially bounded function.

### Corollary 1

*Let* $a>0$, $b>0$, $b^{2}(\theta -\tau)^{2}>4(a-b)$, $\tau <\theta$,

2.11 $$\begin{aligned}& b(\theta -\tau)\theta \biggl\{ 1+\frac{2}{b^{2}(\theta -\tau)^{2}+\sqrt{b ^{2}(\theta -\tau)^{2}-4(a-b)}} \\& \quad {}\times \biggl\{ \frac{b(\theta -\tau)-\sqrt{b^{2}(\theta - \tau)^{2}-4(a-b)}}{b(\theta -\tau)+ \sqrt{b^{2}(\theta -\tau)^{2}-4(a-b)}} \biggr\} ^{\frac{b(\theta - \tau)-\sqrt{b^{2}(\theta -\tau)^{2}-4(a-b)}}{\sqrt{b^{2}(\theta -\tau)^{2}-4(a-b)}}} \biggr\} \\& \quad {}+(a-b)\tau \frac{4}{b(\theta -\tau)+ \sqrt{b^{2}(\theta -\tau)^{2}-4(a-b)}} \\& \quad {}\times \biggl\{ \frac{b(\theta -\tau)- \sqrt{b^{2}(\theta -\tau)^{2}-4(a-b)}}{b(\theta -\tau)+\sqrt{b ^{2}(\theta -\tau)^{2}-4(a-b)}} \biggr\} ^{\frac{b(\theta -\tau)-\sqrt{b ^{2}(\theta -\tau)^{2}-4B}}{2\sqrt{b^{2}(\theta -\tau)^{2}-4B}}}< 1, \end{aligned}$$

*then the Cauchy function* $C(t,s)$ *of equation* (2.9) *and the fundamental system* $x_{1}(t)$, $x_{2}(t)$ *of equation* (2.9), (2.10) *satisfy an exponential estimate*.

### Example 1

Let us set $a=\sqrt{5}+0.01$, $b=\sqrt{5}$, $\theta -\tau =0.1$. In this case we have $b^{2}(\theta -\tau)^{2}>4(a-b)$ and condition (2.11) is fulfilled if $\theta <1.876$.

### Corollary 2

*Let* $a>0$, $b>0$, $b^{2}(\theta -\tau)^{2}=4(a-b)$, $\tau <\theta$,

2.12 $$ b(\theta -\tau)\theta \biggl\{ 1+\frac{b(\theta -\tau)}{4}- \biggl( 1- \frac{b( \theta -\tau)}{4} \biggr) \frac{1}{e^{2}} \biggr\} +\tau \frac{4(a-b)}{b( \theta -\tau)e}< 1, $$

*then the Cauchy function* $C(t,s)$ *of equation* (2.9) *and the fundamental system* $x_{1}(t)$, $x_{2}(t)$ *of equation* (2.9), (2.10) *satisfy an exponential estimate*.

### Example 2

Let us set $a=2.01$, $b=2$, $\theta -\tau =0.1$. In this case we have $b^{2}(\theta -\tau)^{2}=4(a-b)$ and condition (2.12) is fulfilled if $\theta <4$.

### Corollary 3

*Let* $a>0$, $b>0$, $b^{2}(\theta -\tau)^{2}<4(a-b)$, $\tau <\theta$,

2.13 $$\begin{aligned}& b\theta (\theta -\tau) \biggl\{ \frac{2\exp [ -\frac{b(\theta - \tau)}{\sqrt{4(a-b)-b^{2}(\theta -\tau)^{2}}} ( \pi +2\arctan \frac{\sqrt{4(a-b)-b^{2}(\theta -\tau)^{2}}}{b(\theta -\tau)} ) ] }{1-\exp [ -\frac{b}{ \sqrt{4(a-b)-b^{2}(\theta -\tau)^{2}}}\pi ] }+1 \biggr\} \\& \quad {} +2\sqrt{(a-b)}\tau \frac{\exp [ -\frac{b(\theta -\tau)}{\sqrt{4(a-b)-b ^{2}(\theta -\tau)^{2}}} ( \pi +\arctan \frac{\sqrt{4(a-b)-b ^{2}(\theta -\tau)^{2}}}{b(\theta -\tau)} ) ] }{1-\exp [ -\frac{b(\theta -\tau)}{ \sqrt{4(a-b)-b^{2}(\theta -\tau)^{2}}}\pi ] }< 1, \end{aligned}$$

*then the Cauchy function* $C(t,s)$ *of equation* (2.9) *and the fundamental system* $x_{1}(t)$, $x_{2}(t)$ *of equation* (2.9), (2.10) *satisfy an exponential estimate*.

### Example 3

Let us set $a=2$, $b=1.99$, $\theta -\tau =0.1$. In this case we have $b^{2}(\theta -\tau)^{2}<4(a-b)$ and condition (2.13) is fulfilled if $\theta <0.23$.

### Remark 1

Results as regards an exponential stability of equation (2.9), only in the case $\tau =0$, were obtained in [9–11], but as far as we know, there are no other results in the case of positive delay *τ* and oscillating solutions to equation (2.9).

## Estimates of integrals of the Cauchy functions for auxiliary equations

Let us consider the following auxiliary equation:

3.1 $$ x^{\prime \prime }(t)+Ax^{\prime }(t)+Bx(t)=0,\quad t\in [ 0,+ \infty), $$

where *A*, *B* are positive constants. Denote by $W(t,s)$ the Cauchy function of equation (3.1) according to the following rule: for every fixed *s* the function $W(t,s)$, as a function of the variable *t*, satisfies the equation (3.1) and the initial conditions

3.2 $$ x(s)=0,\qquad x^{\prime }(s)=1. $$

For equation (3.1) with constant coefficients, we can construct the Cauchy function $W(t,s)$ and get the following integrals:

3.3 $$ \int _{0}^{t} \bigl\vert W(t,s) \bigr\vert \,ds,\qquad \int _{0}^{t} \bigl\vert W_{t}^{\prime }(t,s) \bigr\vert \,ds\quad \text{and}\quad \int _{0}^{t} \bigl\vert W_{tt}^{\prime \prime }(t,s) \bigr\vert \,ds, \quad 0\leq t< +\infty. $$

Consider all possible cases: (1) $A^{2}>4B$, (2) $A^{2}=4B$, (3) $A^{2}<4B$.

### Lemma 1

*Let* $A>0$, $B>0$, $A^{2}>4B$, *then*

3.4 $$\begin{aligned}& \limsup_{t\rightarrow \infty } \int _{0}^{t} \bigl\vert W(t,s) \bigr\vert \,ds =\frac{1}{B}, \end{aligned}$$

3.5 $$\begin{aligned}& \limsup_{t\rightarrow \infty } \int _{0}^{t} \bigl\vert W_{t}^{ \prime }(t,s) \bigr\vert \,ds=\frac{4}{A+\sqrt{A^{2}-4B}} \biggl\{ \frac{A-\sqrt{A^{2}-4B}}{A+\sqrt{A^{2}-4B}} \biggr\} ^{\frac{A-\sqrt{A ^{2}-4B}}{2\sqrt{A^{2}-4B}}}, \end{aligned}$$

3.6 $$\begin{aligned}& \limsup_{t\rightarrow \infty } \int _{0}^{t} \bigl\vert W_{tt} ^{\prime \prime }(t,s) \bigr\vert \,ds=\frac{2}{A+ \sqrt{A^{2}-4B}} \biggl\{ \frac{A-\sqrt{A^{2}-4B}}{A+ \sqrt{A^{2}-4B}} \biggr\} ^{\frac{A-\sqrt{A^{2}-4B}}{ \sqrt{A^{2}-4B}}}. \end{aligned}$$

### Lemma 2

*Let* $A>0$, $B>0$, $A^{2}=4B$, *then*

3.7 $$\begin{aligned}& \limsup_{t\rightarrow \infty } \int _{0}^{t} \bigl\vert W(t,s) \bigr\vert \,ds =\frac{1}{B}, \end{aligned}$$

3.8 $$\begin{aligned}& \limsup_{t\rightarrow \infty } \int _{0}^{t} \bigl\vert W_{t}^{ \prime }(t,s) \bigr\vert \,ds=\frac{4}{Ae}, \end{aligned}$$

3.9 $$\begin{aligned}& \limsup_{t\rightarrow \infty } \int _{0}^{t} \bigl\vert W_{tt} ^{\prime \prime }(t,s) \bigr\vert \,ds =1+\frac{A}{4}- \biggl( 1- \frac{A}{4} \biggr) \frac{1}{e^{2}}. \end{aligned}$$

### Lemma 3

*Let* $A>0$, $B>0$, $A^{2}<4B$, *then*

3.10 $$\begin{aligned}& \limsup_{t\rightarrow \infty } \int _{0}^{t} \bigl\vert W(t,s) \bigr\vert \,ds =\frac{1}{B}\frac{1+\exp ( -\frac{\pi A}{\sqrt{4B-A ^{2}}} ) }{1-\exp ( -\frac{\pi A}{\sqrt{4B-A^{2}}} ) }, \end{aligned}$$

3.11 $$\begin{aligned}& \limsup_{t\rightarrow \infty } \int _{0}^{t} \bigl\vert W_{t}^{ \prime }(t,s) \bigr\vert \,ds=\frac{2}{\sqrt{B}}\frac{\exp [ -\frac{A}{\sqrt{4B-A^{2}}} ( \pi +\arctan \frac{\sqrt{4B-A ^{2}}}{A} ) ] }{1-\exp [ -\frac{A}{\sqrt{4B-A^{2}}} \pi ] }, \end{aligned}$$

3.12 $$\begin{aligned}& \limsup_{t\rightarrow \infty } \int _{0}^{t} \bigl\vert W_{tt} ^{\prime \prime }(t,s) \bigr\vert \,ds=\frac{2\exp [ -\frac{A}{\sqrt{4B-A ^{2}}} ( \pi +2\arctan \frac{\sqrt{4B-A^{2}}}{A} ) ] }{1-\exp [ -\frac{A}{\sqrt{4B-A^{2}}}\pi ] }. \end{aligned}$$

The proofs of these lemmas can be found in [21], Lemmas 3.1-3.3.

## Proofs of main theorems

The following result, which we formulate here for equation (1.1), is known as the Bohl-Perron theorem.

### Lemma 4

*Suppose the solution of equation* (1.1) *with the zero initial conditions*,

$$x(\xi)=0, \quad \xi \leq 0, $$

*is bounded on* $[0,\infty)$ *for any essentially bounded on the semi*-*axes right*-*hand side* *f*. *Then equation* (1.3) *is uniformly exponentially stable*.

Let us consider the ordinary differential equation

4.1 $$ x^{\prime \prime }(t)+Ax^{\prime }(t)+Bx(t)=z(t), \quad t\in [ 0,+ \infty), $$

with constant positive coefficients *A* and *B*.

It is clear that the solution of equation (4.1) which satisfies the initial conditions

4.2 $$ x(0)=0,\qquad x^{\prime }(0)=0, $$

can be written in the form

4.3 $$ x(t)= \int _{0}^{t}W(t,s)z(s)\,ds, $$

where $W(t,s)$ is the Cauchy function of equation (4.1). Its derivatives are the following:

4.4 $$ x^{\prime }(t)= \int _{0}^{t}W_{t}^{\prime }(t,s)z(s) \,ds,\qquad x^{\prime \prime }(t)= \int _{0}^{t}W_{tt}^{\prime \prime }(t,s)z(s) \,ds+z(t). $$

Let us denote

4.5 $$\begin{aligned}& \Vert W\Vert =\limsup_{t\rightarrow \infty } \int _{0}^{t} \bigl\vert W(t,s) \bigr\vert \,ds, \end{aligned}$$

4.6 $$\begin{aligned}& \bigl\Vert W_{t}^{\prime } \bigr\Vert =\limsup _{t\rightarrow \infty } \int _{0}^{t} \bigl\vert W_{t}^{\prime }(t,s) \bigr\vert \,ds,\qquad \bigl\Vert W_{tt}^{\prime \prime } \bigr\Vert = \limsup_{t\rightarrow \infty } \int _{0}^{t} \bigl\vert W_{tt} ^{\prime \prime }(t,s) \bigr\vert \,ds. \end{aligned}$$

### Theorem 4

*Let* $A>0$, $B>0$, $\tau_{i}(t)\leq \theta_{i}(t)$ *and the following inequality be fulfilled*:

4.7 $$\begin{aligned}& \sum_{i=1}^{m} \bigl\vert A_{i}^{0} \bigr\vert \theta_{i}^{ \ast } \bigl\{ \bigl\Vert W_{tt}^{\prime \prime } \bigr\Vert +1 \bigr\} +\sum _{i=1}^{m} \bigl\vert \Delta A_{i}(t) \bigr\vert \bigl\Vert W _{t}^{\prime } \bigr\Vert \\& \quad +\sum_{i=1}^{m} \bigl\vert B_{i}^{0} \bigr\vert \tau_{i}^{ \ast } \bigl\Vert W_{t}^{\prime } \bigr\Vert +\sum _{i=1}^{m} \bigl\vert \Delta B_{i}(t) \bigr\vert \Vert W\Vert < 1, \end{aligned}$$

*then the Cauchy function* $C(t,s)$ *of equation* (1.1) *and the fundamental system* $x_{1}(t)$, $x_{2}(t)$ *of equation* (1.3), (1.2) *satisfy an exponential estimate*.

### Remark 2

It is clear from inequality (4.7) that in the case, when the coefficients $a_{i}(t)-b_{i}(t)$ and $b_{i}(t)(\theta_{i}(t)-\tau_{i}(t))$ ($1=1,\ldots,m$) are close to constants, the second and fourth terms are small, and in the case of small delays $\theta_{i}(t)$ and $\tau_{i}(t) $ ($1=1,\ldots,m$), the first and the third terms are small. We can draw the conclusion that in this case equation (1.1) preserves an exponential stability of equation (2.1).

### Proof

Consider the equation

4.8 $$\begin{aligned}& x^{\prime \prime }(t)+\sum_{i=1}^{m}a_{i}(t)x \bigl(t-\tau_{i}(t) \bigr)- \sum_{i=1}^{m}b_{i}(t)x \bigl(t-\theta_{i}(t) \bigr)=f(t), \quad t\in [ 0,+\infty), \end{aligned}$$

4.9 $$\begin{aligned}& x(\xi)=0,\quad \text{for } \xi < 0. \end{aligned}$$

Let us rewrite equation (4.8) in the following forms:

4.10 $$\begin{aligned}& x^{\prime \prime }(t)+\sum_{i=1}^{m} \bigl[a_{i}(t)-b_{i}(t) \bigr]x \bigl(t- \tau_{i}(t) \bigr)+\sum_{i=1}^{m}b_{i}(t) \bigl[x \bigl(t-\tau_{i}(t) \bigr)-x \bigl(t-\theta_{i}(t) \bigr) \bigr] =f(t), \\& \quad t\in [ 0,+\infty), \end{aligned}$$

and

4.11 $$\begin{aligned}& x^{\prime \prime }(t)+\sum_{i=1}^{m}b_{i}(t) \int _{t-\theta_{i}(t)}^{t-\tau_{i}(t)}x^{\prime }(s)\,ds+\sum _{i=1}^{m} \bigl[a_{i}(t)-b_{i}(t) \bigr]x \bigl(t-\tau_{i}(t) \bigr) =f(t), \\& \quad t\in [ 0,+\infty), \end{aligned}$$

where

4.12 $$ x(\xi)=x^{\prime }(\xi)=0,\quad \text{for } \xi < 0. $$

We have to prove that, for every essentially bounded function $f(t)$, the solution $x(t)$ is also bounded on the semiaxis $t\in [ 0,+\infty)$. To prove exponential stability of (4.8) we assume the existence of an unbounded solution $x(t)$ and demonstrate that this is impossible.

There exist measurable delay functions $\eta_{i},\tau_{i}(t)\leq \eta _{i}(t)\leq \theta_{i}(t)$ such that equality (4.11) can be written as

4.13 $$\begin{aligned}& x^{\prime \prime }(t)+\sum_{i=1}^{m}b_{i}(t) \bigl[\theta_{i}(t)-\tau _{i}(t) \bigr]x^{\prime } \bigl(t-\eta_{i}(t) \bigr)+\sum_{i=1}^{m} \bigl[a_{i}(t)-b _{i}(t) \bigr]x \bigl(t- \tau_{i}(t) \bigr)=f(t), \\& \quad t\in [ 0,+\infty), \end{aligned}$$

and then in the form

4.14 $$ x^{\prime \prime }(t)+\sum_{i=1}^{m}A_{i}(t)x^{\prime } \bigl(t- \eta_{i}(t) \bigr)+\sum_{i=1}^{m}B_{i}(t)x \bigl(t-\tau_{i}(t) \bigr)=f(t), $$

where $A_{i}(t)= b_{i}(t)[\theta_{i}(t)-\tau_{i}(t)]$ and $B_{i}(t)= a_{i}(t)-b_{i}(t)$. Let us rewrite it in the form

4.15 $$\begin{aligned}& x^{\prime \prime }(t)+Ax^{\prime }(t)-Ax^{\prime }(t)+\sum _{i=1}^{m}A_{i}(t)x^{\prime } \bigl(t-\eta_{i}(t) \bigr)+Bx(t)-Bx(t)+\sum _{i=1} ^{m}B_{i}(t)x \bigl(t- \tau_{i}(t) \bigr) \\& \quad =f(t), \end{aligned}$$

hence

4.16 $$\begin{aligned}& x^{\prime \prime }(t)+Ax^{\prime }(t)+Bx(t)=\sum_{i=1}^{m}A _{i}^{0} \int _{t-\eta_{i}(t)}^{t}x^{\prime \prime }(s)\,ds+ \sum _{i=1}^{m}\Delta A_{i}(t)x^{\prime } \bigl(t-\eta_{i}(t) \bigr) \\& \quad {}+\sum_{i=1}^{m}B_{i}^{0} \int _{t-\tau_{i}(t)}^{t}x^{ \prime }(s)\,ds+\sum _{i=1}^{m}\Delta B_{i}(t)x \bigl(t- \tau_{i}(t) \bigr)+f(t),\quad t\in [ 0,\infty), \end{aligned}$$

where

4.17 $$ x(\xi)=x^{\prime }(\xi)=x^{\prime \prime }(\xi)=0,\quad \text{for } \xi < 0, $$

and $\Delta A_{i}(t)$, $A_{i}^{0}$, $\Delta B_{i}(t)$, $B_{i}^{0}$ are defined by equations (2.2)-(2.4).

Let us make a so-called *W*-transform, substituting $x(t)=\int _{0}^{t}W(t,s)z(s)\,ds$, where $z\in L_{\infty }$ ($L_{\infty }$ is the space of essentially bounded functions $z:[0,\infty)\rightarrow (- \infty,+\infty)$), into equation (4.16). It is clear that the derivatives $x^{\prime }(t)$ and $x^{\prime \prime }(t)$ are defined by equalities (4.4). We get the following equation:

4.18 $$ z(t)=(Kz) (t)+f(t), $$

where the operator $K:L_{\infty }\rightarrow L_{\infty }$ is defined by the equality

4.19 $$\begin{aligned} (Kz) (t) =&\sum_{i=1}^{m}A_{i}^{0} \sigma \bigl(t-\eta_{i}(t) \bigr) \int _{t-\eta_{i}(t)}^{t} \biggl\{ \int _{0}^{s}W_{ss}^{\prime \prime }(s, \xi)z(\xi)\,d\xi +z(s) \biggr\} \,ds \\ &{}+\sum_{i=1}^{m}\Delta A_{i}(t) \sigma \bigl(t-\eta_{i}(t) \bigr) \int _{0}^{t-\eta_{i}(t)}W_{t}^{\prime } \bigl(t- \eta_{i}(t),s \bigr)z(s)\,ds \\ &{} +\sum_{i=1}^{m}B_{i}^{0} \sigma \bigl(t-\tau_{i}(t) \bigr) \int _{t-\tau_{i}(t)}^{t} \int _{0}^{s}W_{s}^{\prime }(s, \xi)z(\xi)\,d\xi \,ds \\ &{}+ \sum_{i=1}^{m}\Delta B_{i}(t)\sigma \bigl(t-\tau_{i}(t) \bigr) \int _{0}^{t-\tau_{i}(t)}W \bigl(t-\tau_{i}(t),s \bigr)z(s)\,ds, \end{aligned}$$

and

4.20 $$ \sigma (t)=\textstyle\begin{cases} 1,& t\geq 0, \\ 0,& t< 0. \end{cases} $$

The inequality (4.7) implies that the norm $\Vert K\Vert $ of the operator $K:L_{\infty }\rightarrow L _{\infty }$ is less than one. In this case, there exists the bounded operator $(I-K)^{-1}$. For every bounded right-hand side the solution *z* of equation (4.18) is bounded.

In the case $A>0$ and $B>0$, the Cauchy function $W(t,s)$ and its derivative $W_{t}^{\prime }(t,s)$ satisfy exponential estimates. The boundedness of the solution *x* of equation (1.1) and its derivative $x^{\prime }$ follow now from the boundedness of *z*.

We have got a contradiction with our assumption that the solution $x(t)$ is unbounded on the semiaxis.

Thus solutions $x(t)$ are bounded on the semiaxis for a bounded right-hand side $f(t)$. Then by Lemma 4 the Cauchy function $C(t,s)$ of equation (1.1) and the solutions $x_{1}$ and $x_{2}$ satisfy the exponential estimates.

This completes the proof of Theorem 4. □

To prove Theorems 1-3 we set the norms of $\Vert W\Vert $, $\Vert W_{t}^{\prime }\Vert $ and $\Vert W_{tt}^{\prime \prime }\Vert $ obtained in Lemmas 1-3 into Theorem 4.

The proofs of Corollaries 1-3 are results of substitution of $\Vert W\Vert $, $\Vert W_{t} ^{\prime }\Vert $ and $\Vert W_{tt}^{\prime \prime }\Vert $ into Theorems 1-3, when we take into account that $A=b(\theta -\tau)$, $B=a-b$, $\Delta A=\Delta B=0$.

## Conclusion

In this paper to obtain exponential stability conditions we use the substitution

$$x(t)=(Wz) (t):= \int_{t_{0}}^{t} Y(t,s) z(s)\,ds, $$

where $Y(t,s)$ is the fundamental function of exponentially stable autonomous ordinary differential equation of the second order and then analyze the operator equation

$$z=Tz+f $$

in some functional Banach spaces on semi-axes. This method is usually called the W-method and used in many problems for FDE such as stability, oscillation and non-oscillation, and boundary value problems. To apply this method in our research we need integral estimations of a fundamental function and its first and second derivative. Such estimates were obtained recently and allow us to obtain here new stability results for a wide class of delay differential equations of the second order. In particular we obtain new stability results for delay equations with positive and negative coefficients. Such equations without delay are unstable so the results obtained here one can use to stabilize mathematical models described by ordinary differential equations of the second order.

## Discussion and some topics for future research

The idea of applications of W-transform for second order delay differential equations first appeared in paper [22]. Lemmas 1 and 2 were taken from [21]. All other results in the paper are new and have not been published before.

Explicit integral estimates of the fundamental function and its derivatives we obtain here only for the simplest equation: ordinary differential equation with constant coefficients. It is interesting to obtain such estimates for the ordinary differential equation with variable coefficients or for delay differential equation with constant coefficients. It will allow one to improve the results obtained in this paper.

We did not study here nonlinear equations. It is interesting to consider the W-transformation method for nonlinear equations such that

$$\ddot{x}(t)+f \bigl(t,\dot{x} \bigl(r(t) \bigr) \bigr)+G \bigl(t,x \bigl(h(t) \bigr) \bigr)=0. $$

The next problem is to apply the W-transformation method for the instability of delay differential equations of the second order.

We suppose that the W-transformation method can also be applied for a vector delay differential equation

$$\ddot{X}(t)+A(t)\dot{X} \bigl(g(t) \bigr)+B(t)X \bigl(h(t) \bigr)=0, $$

where *A* and *B* are $n\times n$ matrix-functions. To apply this method it is necessary to obtain explicit integral estimations for the fundamental matrix and its derivative of the following vector ordinary differential equation with constant coefficients:

$$\ddot{X}(t)+A\dot{X}(t)+BX(t)=0. $$
